# Case Report: Experience and Lesson From Postpartum Ruptured Abdominal Aortic Aneurysm

**DOI:** 10.3389/fmed.2021.729099

**Published:** 2022-01-20

**Authors:** Yumei Wang, Di Tian, Liu Yang, Qian Zhang, Guangyao Qin, Xinghui Liu

**Affiliations:** ^1^Department of Obstetrics and Gynecology, West China Second University Hospital, Sichuan University, Chengdu, China; ^2^Department of Health Care, Chengdu Women's and Children's Central Hospital, School of Medicine, University of Electronic Science and Technology of China, Chengdu, China; ^3^Key Laboratory of Birth Defects and Related Diseases of Women and Children, Ministry of Education, West China Second University Hospital, Sichuan University, Chengdu, China; ^4^Department of Obstetrics and Gynecology, Pengzhou Maternal and Child Health Care Hospital, Chengdu, China

**Keywords:** abdominal aortic aneurysm, pregnancy, postpartum, neurofibromatosis, case report

## Abstract

With rapid progression and extremely high mortality brought by the ruptured aneurysm, abdominal aortic aneurysm is one of the most dangerous diseases in the field of vascular surgery. The incidence of abdominal aortic aneurysms in pregnant women is low. Because of the insidious nature of the disease, it is not easily recognized and can be easily misdiagnosed by obstetricians, which can lead to serious adverse outcomes. In this study, we aim to draw attention to this disease and raise awareness among obstetricians about this disease in pregnant women by retrospectively analyzing a rescue case of ruptured abdominal aortic aneurysm after delivery. By reporting the experience and lessons learned from this case, we aim to improve preoperative diagnosis and treatment rates, reduce missed diagnosis, and improve the prognostic outcome of patients.

## Introduction

Abdominal aortic aneurysm (AAA) refers to a degenerative abdominal aorta disease in which the main pathological manifestation is a permanent dilation (exceeding 50% of the normal blood vessel or diameter ≥30 mm) of a segment of the abdominal aorta (1). The progression of AAA is the swelling of the aneurysm until it ruptures, and before that AAA is usually asymptomatic. Once it ruptures spontaneously, the patient's circulation rapidly collapses. The disease is critical, with an overall mortality rate of 80%−90%, and mortality rate can still reach 50–70% even if a patient receives medical treatment in time (2–4). Compared with non-pregnant and non-puerperal women, pregnant and puerperal women are faced with a significantly higher risk of vascular events, including rare vascular events such as ruptured aortic aneurysms (5, 6). However, case studies about ruptured AAA in pregnant and puerperal women remain limited. This paper reports the case of a postpartum ruptured AAA.

## Case Description

The patient, a 41-year-old woman who had her last menstruation on January 15, 2020, did not receive regular obstetric examination during pregnancy but was diagnosed with gestational diabetes mellitus at 26 weeks of pregnancy. She controlled blood glucose through diet and exercise, but the effectiveness of these measures was unclear because of irregular blood glucose monitoring. According to her description, she had a heritable disease that showed extensive fibromas on the skin of the entire body and her father had similar physical signs. But the disorder was not diagnosed definitely. She had previously delivered a live baby girl via vaginal delivery in 2001.

Due to a labor precursor, the patient was admitted to the county level hospital at 38 weeks of pregnancy. At that time, her vital signs were stable, and routine laboratory examination showed no significant abnormalities. However, physical examination revealed extensive and irregular fibromas on the skin throughout the body, the largest of which was ~1 cm in diameter, with no redness, swelling, ulceration, or pain. At the onset of labor, she attempted vaginal delivery but ultimately delivered a live male baby by cesarean section due to stagnation during the active phase of labor. During the operation, no abnormalities were found in the uterus, bilateral adnexa, or pelvic cavity. Blood loss was 650 ml within 24 h after delivery, including 500 ml during the surgery. Postoperative vital signs were normal. The hemoglobin level was 115 g/L on the first postoperative day, and defecation was normal.

However, the patient suddenly developed syncope at dusk with urinary incontinence on the fourth postoperative day, when she was changed from supine to a semi-supine position. Immediately examined by the obstetrician, the patient's heart rate was 100 beats per minute, but her blood pressure could not be obtained by routine measurements. She was not breathing spontaneously and exhibited systemic cyanosis. The left and right pupil was 0.2 and 0.3 cm in diameter, respectively. Auscultation did not reveal any significant cardiac or pulmonary abnormalities, but the carotid artery pulsation was weak. Within a minute, she was given a series of treatment, including continuous pressure oxygenation with an airbag mask, ECG monitoring, indwelling catheterization and anti-shock treatment. Besides, a multidisciplinary team was invited for an emergency consultation, and the neurosurgeon noted that this patient had extensive and irregular fibromas throughout her body, which indicated that she may have the genetic disorder neurofibromatosis.

Five minutes later, the patient responded, but she was agitated and complained of pain in her low back. At that time, her body temperature was 35.8°C, heart rate was 108 beats per minute, blood pressure was 82/54 mmHg, respiratory rate was 25 breaths per minute, and blood oxygen saturation was 81%. The patient presented cyanosis, a soft abdomen without distention, good uterine contractions, and a small amount of vaginal bleeding. After receiving active but ineffective anti-shock treatment, the patient's blood pressure continued to decrease progressively, falling to 58/40 mmHg after 10 min. At this time, her heart rate was 120 beats per minute and blood oxygen saturation was 81%. B-mode ultrasound showed the collapse of the inferior vena cava, but no pleural effusion, no pericardial tamponade, and no pulmonary embolism was observed. Therefore, pulmonary embolism was not considered. Hemoglobin was 70 g/L and blood glucose was 6.2 mmol/L. Blood gas analysis indicated a pH of 6.89 and ${\text{HCO}}_{3}^{-}$ level of 6.1 mmol/L. we quickly initiated a mass transfusion program and corrected the acidosis.

**Figure 1:**
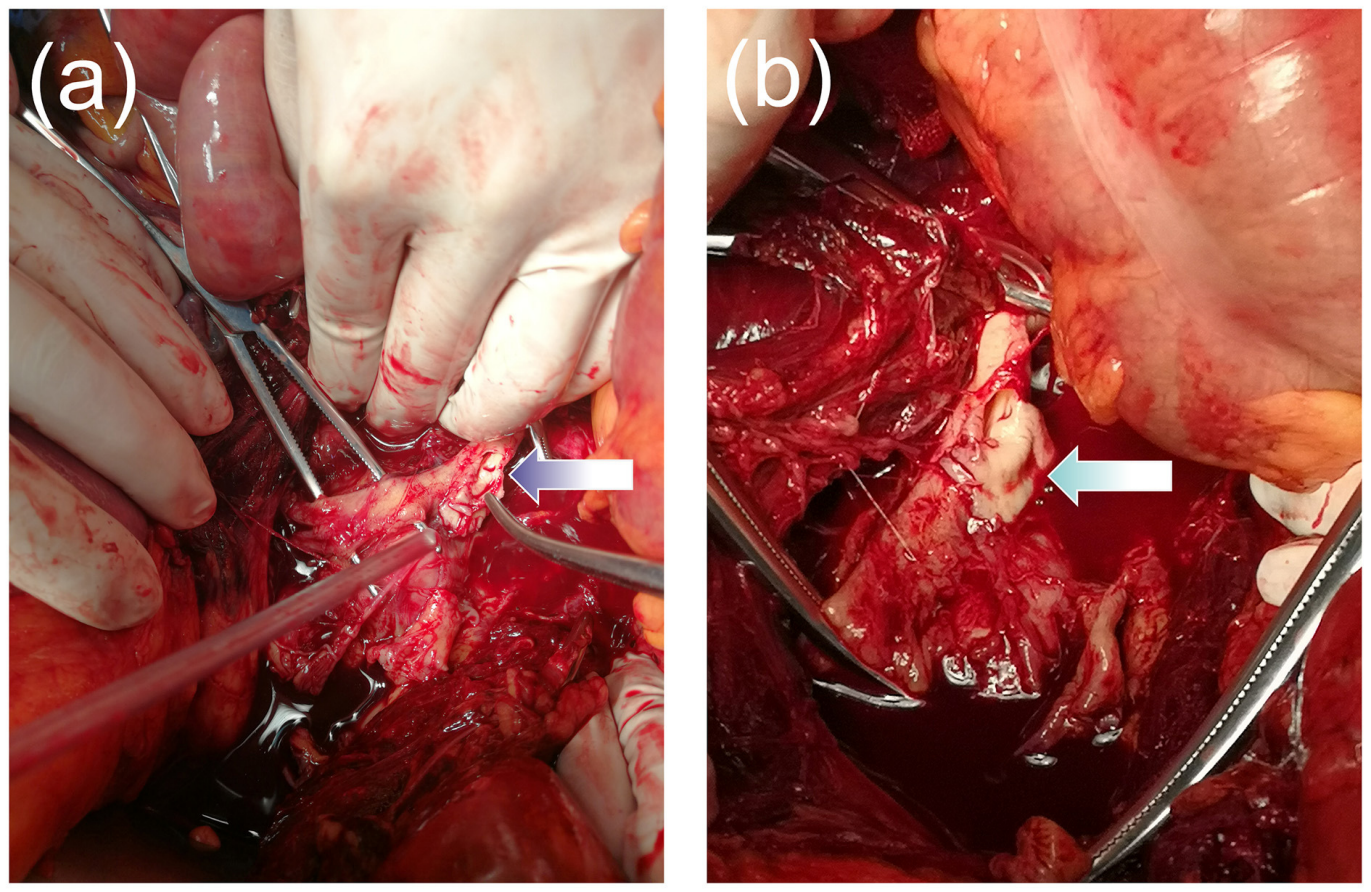
**(a,b)** Image showing the aneurysm rupture at the abdominal aortic bifurcation. The wall of the aorta was severely lacerated with a thin and irregular rupture.

Approximately half an hour later, the patient went into a coma again, at which time her heart rate was 125 beats per minute, blood pressure was 80/62 mmHg, and oxygen saturation was 80%. The conjunctiva was slightly pale, the abdomen was soft with no abdominal swelling, the uterine was well contracted, vaginal bleeding was reduced, and no urine was in the urinary bag tube. We immediately intubated her with tracheal intubation and continued positive pressure ventilation to correct the patient's shock state and actively searched for the cause. At the same time, she was continuously monitored by B-mode ultrasound. One hour later, it showed anechoic areas in the liver, kidneys, and the crypt of the right iliac fossa with a maximum anterior-posterior diameter of approximately 4.4 cm. The anechoic areas increased rapidly. We performed abdominal puncture on the patient under ultrasound monitoring immediately and aspirated 5 mL of dark red and noncoagulated blood, at which point we considered the possibility of intra-abdominal hemorrhage and prepared for an urgent exploratory laparotomy. At the same time, the patient was given an aggressive blood transfusion to correct the state of shock. Obstetricians and gynecologists, general surgeons, and vascular surgeons jointly performed a laparotomy for emergency investigation. Upon opening the peritoneum, a large amount of blood and blood clots (estimated volume of 7,000 ml) were found in the abdominal cavity. We saw a huge hematoma under the posterior peritoneum, and a large amount of blood flowed out from the rupture of the posterior peritoneum.The surgeons quickly opened the posterior peritoneum and located the source of the massive bleeding: an abdominal aortic bifurcation rupture, approximately 5 cm in length an severely lacerated with a thin and irregular rupture wall, was found (Figure 1). We quickly cross-clamped the abdominal aorta in an attempt to repair the rupture, but it was considered to be irreparable. The patient suffered cardiac arrest 40 min after the start of the operation and was declared dead after all resuscitation attempts failed. The clinical cause of death was considered to be hemorrhagic shock due to AAA rupture.

## Discussion

The onset of AAA is insidious, and the incidence varies by age, sex, and geography. The total population incidence of this disease is close to 5%, with men ~4–6 times more likely to develop AAA than women (7–9). Several studies have suggested that risk factors for its onset included age over 40, smoking, hypertension, aortic dysplasia, Marfan syndrome and autosomal dominant connective tissue disease (10, 11). Atsushi et al. reported a case of spontaneous rupture of a thoracic aortic aneurysm that happened in a pregnant woman with type I neurofibromatosis, an autosomal dominant connective tissue diseaseat, at 30 weeks of gestation (12). Several studies have also suggested that diabetes may be a risk factor for AAA, although this has remained to be proved (13, 14). Besides, pregnancy is a known risk factor for aortic disease, with more than 50% of aneurysm ruptures in younger women occurring during pregnancy, during which time they are faced with a high maternal mortality rate (15). However, the mechanism by which pregnancy induced aortic pathology remains uncertain, but it may be related to changes in hemodynamics and hormone levels during pregnanc, which may lead to changes in the structure of the vascular wall in patients, especially those with a genetic predisposition (16–18).

The patient, in this case, is a 41-year-old puerperal woman with gestational diabetes mellitus and no history of cardiovascular disease. Therefore, the occurrence of an AAA in this patient may be related to these risk factors (puerperium, age, gestational diabetes mellitus). In addition, the patient has a familial genetic disorder that causes extensive fibromas in her skin throughout her body, which is likely to be neurofibromatosis. When pregnant and puerperal women have connective tissue diseases, the interaction of altered hemodynamics, varied hormonal levels, and changes in the structure of vessel wall together increase the risk of maternal aneurysm formation and rupture (5). This is particularly dangerous in late pregnancy and the postpartum period (5), as the hemodynamic changes caused by pregnancy usually return to the normal level within 3–6 months after childbirth (19). In this case, the patient had her onset on the fourth postoperative day, which is consistent with the above-mentioned report (5). In addition, she underwent a cesarean section after a failed vaginal delivery. This may be another factor leading to the rupture of the AAA. Increased body energy and oxygen consumption during vaginal delivery, combined with uterine contractions, pain, tension, increased abdominal pressure, and other stimuli, may have altered the hemodynamics, which may contribute to the rupturing of the aortic aneurysm.

The preoperative diagnosis of AAA in pregnant women is very difficult. Its routine auxiliary examinations include B-mode ultrasound, X-rays, computed tomography angiography, and magnetic resonance imaging. However, due to the low incidence of AAA, routine pregnancy examinations usually do not include tests related to AAA. In addition, exploration of the upper abdomen and abdominal aorta is not routinely performed during cesarean delivery. Besides, symptoms of AAA may also be masked by pregnancy and postoperative pain, which further affects the early diagnosis of the disease. The rate of misdiagnosis of AAA rupture is 32–42%, and only a small portion can be quickly diagnosed (20). Therefore, timely diagnosis and evaluation of AAA rupture are crucial for successful resuscitation. The triad of symptoms associated with AAA rupture is sudden severe abdominal or low back pain, hypotension or shock, and a pulsatile abdominal mass. Similarly, the clinical manifestations of this patient were sudden syncope and shock with low back pain after a change in body position. Despite active anti-shock treatment, the patient's blood pressure continued to fall progressively and the disease developed rapidly. Although the diagnosis was timely, resuscitation was ineffective and the patient eventually died due to severe injury in the abdominal aorta with excessive blood loss.

Based on this case, we propose the following recommendations. First, pregnant and parturient women with multiple high-risk factors, such as advanced age, gestational diabetes mellitus and connective tissue diseases, should be considered to face the risk of developing AAA, and relevant examinations should be conducted to screen for AAA. Second, for post-cesarean patients who developed symptoms of shock with no evidence of abdominal distension or atonic bleed, the vascular cause should be the main priority, such as a ruptured AAA. Therefore, laparotomy should be performed expediently and rapidly, even without imaging. Third, multi-disciplinary consultation should be done for any pregnancy complicated with AAA. Cesarean section is recommended to terminate pregnancy in such patients, and monitoring of the aorta should be strengthened during labor and puerperium.

This case serves to remind obstetricians the necessity of a broad differential diagnosis. In patients exhibiting shock that is inconsistent with the clinical symptoms due to the extent of vaginal bleeding, we should actively investigate the cause of shock and treat it rapidly. Further studies are required to explore methods to identify this disease in the early stage, to improve the success rate of treatment, and to prevent the disease from progressing.

## Data Availability Statement

The raw data supporting the conclusions of this article will be made available by the authors, without undue reservation.

## Ethics Statement

Written informed consent was obtained from the individual(s) for the publication of any potentially identifiable images or data included in this article.

## Author Contributions

LY, QZ, and GQ: analyzed patient data. DT and YW: designed and wrote the paper. XL: revised the manuscript. All authors commented on the manuscript.

## Funding

This work was supported by Sichuan Science and Technology Project of China (2020YFS0488).

## Conflict of Interest

The authors declare that the research was conducted in the absence of any commercial or financial relationships that could be construed as a potential conflict of interest.

## Publisher's Note

All claims expressed in this article are solely those of the authors and do not necessarily represent those of their affiliated organizations, or those of the publisher, the editors and the reviewers. Any product that may be evaluated in this article, or claim that may be made by its manufacturer, is not guaranteed or endorsed by the publisher.
